# Modeling T cell receptor recognition of CD1-lipid and MR1-metabolite complexes

**DOI:** 10.1186/1471-2105-15-319

**Published:** 2014-09-26

**Authors:** Brian G Pierce, Thom Vreven, Zhiping Weng

**Affiliations:** Program in Bioinformatics and Integrative Biology, University of Massachusetts Medical School, 368 Plantation Street, Worcester, MA 01605 USA; University of Maryland Institute for Bioscience and Biotechnology Research, 9600 Gudelsky Drive, Rockville, MD 20850 USA

**Keywords:** TCR, MAIT, CD1d, CD1b, GEM, MHC-like

## Abstract

**Background:**

T cell receptors (TCRs) can recognize diverse lipid and metabolite antigens presented by MHC-like molecules CD1 and MR1, and the molecular basis of many of these interactions has not been determined. Here we applied our protein docking algorithm TCRFlexDock, previously developed to perform docking of TCRs to peptide-MHC (pMHC) molecules, to predict the binding of αβ and γδ TCRs to CD1 and MR1, starting with the structures of the unbound molecules.

**Results:**

Evaluating against TCR-CD1d complexes with crystal structures, we achieved near-native structures in the top 20 models for two out of four cases, and an acceptable-rated prediction for a third case. We also predicted the structure of an interaction between a MAIT TCR and MR1-antigen that has not been structurally characterized, yielding a top-ranked model that agreed remarkably with a characterized TCR-MR1-antigen structure that has a nearly identical TCR α chain but a different β chain, highlighting the likely dominance of the conserved α chain in MR1-antigen recognition. Docking performance was improved by re-training our scoring function with a set of TCR-pMHC complexes, and for a case with an outlier binding mode, we found that alternative docking start positions improved predictive accuracy. We then performed unbound docking with two mycolyl-lipid specific TCRs that recognize lipid-bound CD1b, which represent a class of interactions that is not structurally characterized. Highly-ranked models of these complexes showed remarkable agreement between their binding topologies, as expected based on their shared germline sequences, while differences in residue-level interactions with their respective antigens point to possible mechanisms underlying their distinct specificities.

**Conclusions:**

Together these results indicate that flexible docking simulations can provide accurate models and atomic-level insights into TCR recognition of MHC-like molecules presenting lipid and other small molecule antigens.

**Electronic supplementary material:**

The online version of this article (doi:10.1186/1471-2105-15-319) contains supplementary material, which is available to authorized users.

## Background

T cell receptors (TCRs) display remarkable versatility in their ability to specifically recognize a wide array of structurally and chemically diverse antigens. This is highlighted by a number of studies showing that, in addition to well-characterized recognition of peptide-major histocompatibility complex (pMHC) [1], many TCRs engage MHC-like molecules CD1, which present a variety of lipid antigens [2], and MR1, which presents vitamin B metabolites [3]. At the sequence level, these TCRs can be restricted or diverse in their germ-line chain usage, depending in part on the T cell type, antigen, and antigen-presenting molecule [2].

The understanding of the molecular basis of TCR interactions with CD1 and MR1 has been greatly advanced by a number of crystallographic studies that have elucidated the interface sites, key contacts, and binding modes of several of these complexes. This includes type I and type II natural killer T cell (NKT) TCR interactions with CD1d-lipid [4–6], mucosal-associated invariant T cell (MAIT) TCR interfaces with unliganded [7] and ligand-bound MR1 [8], and recent studies of two γδ TCRs bound to CD1d-lipid [9, 10]. While these have provided a clear view of the antigen recognition underlying several of the invariant and diverse T cell subsets, owing to their variety and the effort required in experimental structure determination, the crystal structures of a number of key complexes have not yet been solved.

Here we describe the adaptation of the TCR docking algorithm, TCRFlexDock, previously shown to produce highly accurate TCR-pMHC structural predictions [11], to predict complexes of TCRs with CD1 and MR1 with antigen (Ag). We assessed unbound docking performance using four known structures of TCRs with CD1d, showing that for most cases accurate models were produced. We also predicted the complex between a MAIT TCR and MR1-Ag, an interaction that has not yet been structurally described, and assessing models against a related MAIT TCR bound to MR1-Ag, found strong agreement between predicted complex and the likely conserved binding mode. Applying TCRFlexDock to predict the structure of two germline-encoded, mycolyl lipid-reactive (GEM) TCRs bound to CD1b, we produced models that provide insight into both their shared sequence features and distinct antigen specificities.

## Results

### Prediction of TCR-CD1d and TCR-MR1 complexes

**Figure 1:**
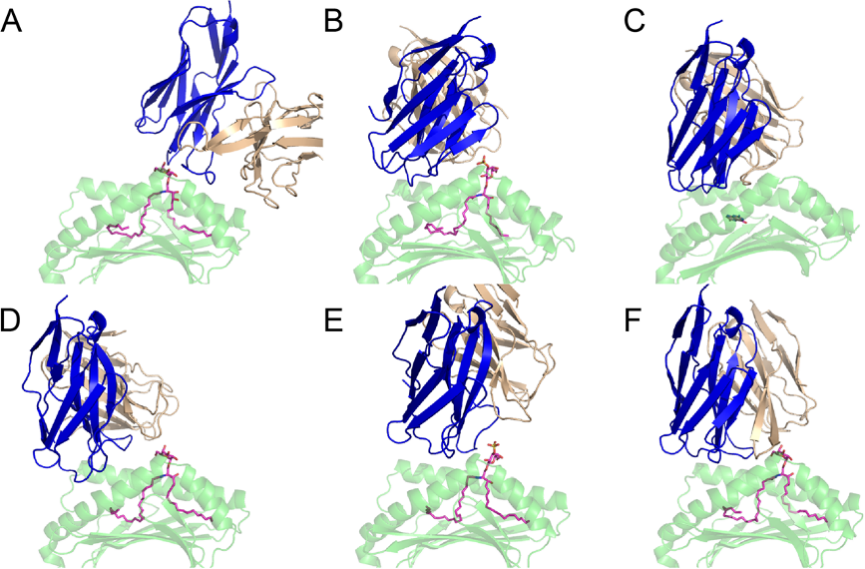
**The structures and starting position of the docking test cases.** Bound structures of **(A)** 3HUJ, **(B)** 4EI5, **(C)** 4L4T, **(D)** 4LHU, **(E)** 4MNG, as well as a representative docking start position **(F)** are shown (test case 3HUJ), with TCRs colored blue (α chain) and gold (β chain), CD1 and MR1 green, and antigens magenta. Not shown are TCR constant domains, as well as the α3 domain and β2m of CD1d and MR1.

Searching for existing unbound and bound TCR and MHC-like protein structures in the Protein Data Bank (PDB) [12], we identified four TCR-CD1d-Ag test cases (Table 1) representing various classes of TCRs (Type I NKT TCR, Type II NKT TCR, and two γδ TCRs). Additional complexes of the same class with essentially identical docking orientations (e.g. Type I NKT TCRs bound to CD1d and other antigens) were not included. We also identified a case with an unbound MAIT TCR (TRAV1-2-TRAJ33-TRBV20 germ line) that binds MR1 presenting the antigen 6FP (6-formyl pterin, a vitamin B metabolite), with a nearly identical sequence to the α chain from a structure of another MAIT TCR (TRAV1-2-TRAJ33-TRBV6 germ line) bound to MR1-6-FP (Table 1); two residues are substituted near the N-term of the CDR3α loop. Given the likely “common mode of MAIT TCR-MR1 docking” [13], supported by additional MAIT TCR complexes with bovine MR1 and TRBV6 TCR variants [14], we evaluated docking predictions of the unbound TRBV20 MAIT TCR to MR1-6FP using the conserved α chain and MR1 in the bound structure, to determine whether the α chain binding conformation is recapitulated by docking, and to predict how the distinctive TRBV20 chain engages MR1-Ag. Collectively, these TCR-CD1d-Ag and TCR-MR1-Ag structures represent a wide variety of docking modes (Figure 1), with greater variability than the TCR-pMHC complexes we considered in our previous work [11].

**Table 1:** **The TCR-CD1d-Ag and TCR-MR1-Ag test cases**

PDB code							
Complex	TCR	Ligand	TCR name	TCR type	Ligand name	Binding RMSD ^1^, Å	Difficulty ^2^
3HUJ [15]	2EYS [16]	1ZT4 [17]	NKT15	Type I NKT	CD1d-α-GalCer	1.04	Rigid
4EI5 [5]	4EI6 [5]	2AKR [18]	XV19	Type II NKT	CD1d-sulfatide	1.08	Medium
4LHU [9]	4LFH [9]	1ZT4 [17]	9C2	γδ	CD1d-α-GalCer	0.90	Rigid
4MNG [10]	4MNH [10]	4MQ7 [10]	DP10.7	γδ	CD1d-sulfatide	0.68	Rigid
4L4T [8] ^3^	4DZB [19] ^3^	4GUP [3]	MAIT	MAIT	MR1-6FP	0.87	Rigid

^1^Backbone atom root-mean-square distance between interface residues in the bound structure and corresponding residues in the unbound structures.

^2^Docking difficulty, based on extent of binding conformational changes [20].

^3^The bound and unbound MAIT TCRs are closely related, with nearly identical α chains; the differing β chains (TRBV6 in 4L4T and TRBV20 in 4DZB) were excluded from RMSD calculations and evaluation of docking predictions.

**Figure 2:**
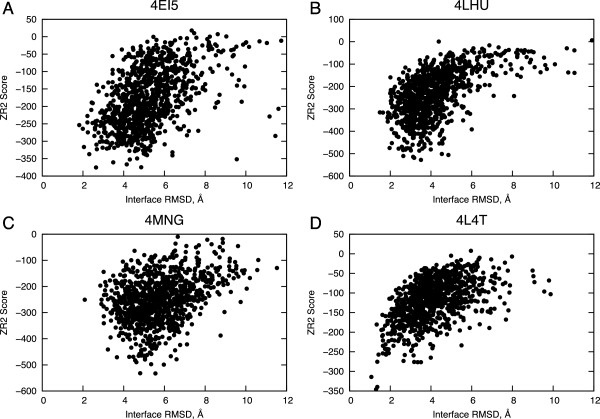
**Binding funnels of TCR-CD1d and TCR-MR1 test cases.** Scores from ZRANK 2 (ZR2) versus interface RMSDs are given for **(A)** 4EI5, **(B)** 4LHU, **(C)** 4MNG, and **(D)** 4L4T, with only residues from the α chain and MAIT being used to calculate interface RMSD for **(D)**.

We performed flexible protein docking using TCRFlexDock to predict the complexes in Table 1 using the unbound TCR and CD1d-Ag/MR1-Ag structures; results are shown in Table 2, with test cases represented by their complex PDB IDs from Table 1. Scores versus interface root-mean-square distances (RMSDs) for cases with hits are shown in Figure 2. We assessed models using CAPRI criteria [21], classifying them as incorrect, acceptable, medium accuracy, or high accuracy, and defined hits, as in our previous study of TCR-pMHC docking [11], as those with medium or high accuracy. For two out of the four CD1d test cases in Table 1 (4EI5 and 4LHU), hits were identified in the top 20 predictions, ranked as in our previous work by ZRANK 2 (ZR2). For test case 4MNG (γδ TCR-CD1d-sulfatide), relatively few hit predictions were generated, and these were not well-ranked (the top hit was ranked 235). Despite its rigid-body classification due to minimal binding conformational changes, this case was challenging due to its atypical docking orientation featuring only the δ TCR chain contacting CD1d-sulfatide [10], resulting in a significant distance from the docking start position as well as a smaller interface area which is unfavorable for the docking scoring function. However, near-hits (acceptable predictions; in parentheses in Table 1) were highly ranked for this case, with a top rank of 10. For test case 3HUJ, no hits were found among the 1000 TCRFlexDock models, which was likely due to its outlier binding mode and distance from the starting docking orientation (over 20 Å ligand RMSD). Performance in this case was improved by employing alternative docking start sites closer to its bound orientation, as noted below.

**Table 2:** **The predictive docking performance starting from the “start1” site**

	Start RMSD, Å					
Test case	Ligand	Interface	Hits ^1^	ZR2 Rank ^2^	ZRT Rank ^2^	T20 RMSD ^3^, Å
3HUJ	21.23	9.67	0	-	-	7.91
4EI5	8.95	3.43	7	6	1	2.21
4LHU	8.22	2.42	45	17	8	1.97
4MNG	13.9	5.06	6	235 (10)	107 (8)	3.22
4L4T^4^	5.77	2.63	38	1	1	1.07

^1^Number of hit predictions among the 1000 models from TCRFlexDock.

^2^Rank of the first hit; for 4MNG, values in parentheses denote the ranks of the first “acceptable” prediction.

^3^Lowest interface root mean square distance from bound in the top 20 predictions, ranked by the ZRT scoring function.

^4^Only TCR α chain and MR1 were used to evaluate these predictions, as unbound and bound TCR β chains differ in sequence.

**Figure 3:**
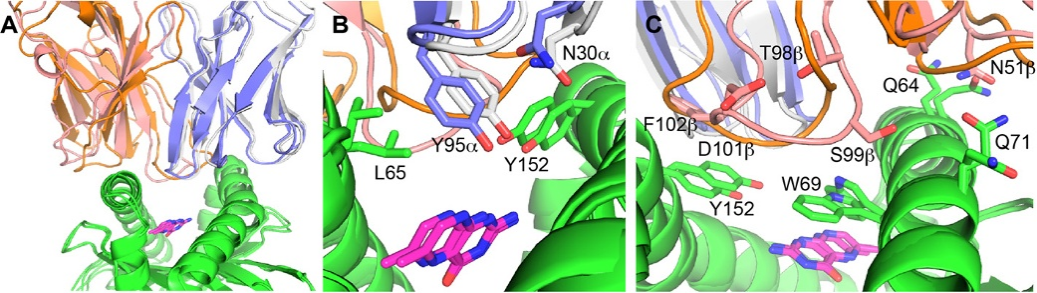
**Predictions of the MAIT TCR-MR1 complex compared with crystal structure of complex with related MAIT TCR.** Shown are the **(A)** complex, **(B)** close-up of the α chain interface with MR1-Ag, and **(C)** close-up of the β chain interface with MR1-Ag, with selected residues show as sticks. Predicted TCR is blue (α chain) and salmon (β chain), TCR from crystal structure (PDB code 4L4T) is gray (α chain) and orange (β chain), MR1 is green, and 6-formyl pterin antigen is magenta. Structures were superposed by MR1 helices.

For the MAIT TCR-MR1-6FP case, the predictions were remarkably consistent with a conserved MAIT TCR α chain binding conformation, with the top-ranked model involving the TRBV20 TCR classified a hit with respect to the bound crystal structure with the TRBV6 TCR, and the third-ranked model having the lowest RMSD (1.07 Å) from the bound structure among all docking models. This model, shown in Figure 3, exhibits nearly perfect matches with TCRα backbone and key side chains in the crystal structure (e.g. hot spot TCR residue Y95α). In contrast, the modeled TRBV20 and crystal structure TRBV6 chains differ somewhat in CDR loop structure and overall orientation. This is consistent with CDR3β swapping experiments between the TRBV20 and TRBV6 MAIT TCRs considered here that ablated MR1-Ag binding, which implied a TRBV germline context dependence for CDR3β loops (“possibly via steric hindrance mechanisms”) [19]. Furthermore there are relatively few favorable side chain contacts between modeled TRBV20 β chain and MR1, in agreement with alanine scanning mutagenesis data [19] that found no individual β chain CDR mutants of this MAIT TCR that significantly altered MR1 recognition.

To investigate whether the TCR CDR loop backbone structure had a significant relationship with overall docking accuracy for these cases, we compared RMSD for modeled versus bound TCR residue backbone atoms (for the subset of TCR residues within 10 Å of CD1-Ag) with rigid-body ligand RMSD for the 1000 docking models of two test cases (Additional file 1: Figure S1). Surprisingly, there was little relationship between these values, indicating that although a bound-like TCR backbone will improve the hit rate of the docking simulation as we previously noted [11], the sampling of CDR loop conformations during docking includes bound-like conformations for both hits and non-hits, and a near-native docking position (i.e. low ligand RMSD) does not necessarily lead to accurately-modeled CDR loops.

### Retraining the TCRFlexDock scoring function

We re-trained the ZRANK scoring function using the previously reported set of TCR-pMHC test cases and docking results [11] to determine whether such an optimized function would improve success on the TCR-CD1 and TCR-MR1 cases in Table 1. The re-weighted function we derived (named ZRT, for ZRANK TCR) indeed led to improved predictive performance in Table 2; though the first hit for case 4MNG was still ranked relatively low, its rank improved (rank 107, versus 235 for ZR2), while the near-hit was ranked 8 by ZRT (versus 10 by ZR2). The new TCR-pMHC derived weights had a lower van der Waals attractive weight relative to the other terms compared with the previous ZRANK function [22], likely due to the lower shape complementarity of TCR-pMHC interactions versus protein-protein interfaces in general [23]. The top-ranked ZRT prediction for test case 4EI5 had a 2.21 Å interface RMSD from native; its structure is shown in Additional file 2: Figure S2. Despite some deviations of the sulfatide antigen head group and CDR3β with respect to the complex crystal structure, the XV19 TCR variable domains as a whole, as well as several key interface side chains, are positioned similarly to those of the known complex.

### Utilizing alternate docking start sites

**Figure 4:**
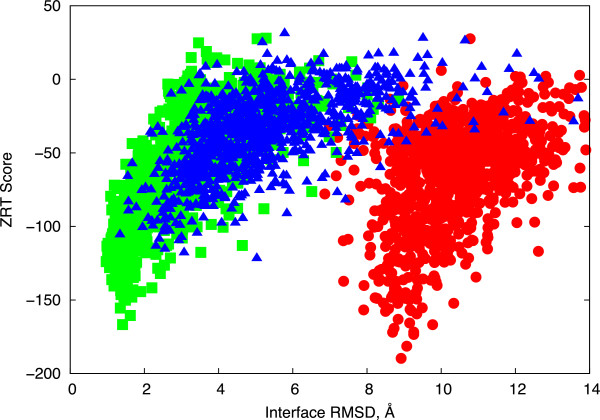
**Binding funnels of the 3HUJ Type I NKT TCR test case.** ZRANK TCR (ZRT) scores versus interface RMSDs are shown for TCRFlexDock simulations starting from the “start1” TCR start position (red circles), “fit” TCR start position (green squares), and shifted “start2” start position (blue triangles).

Considering the distinct binding orientation of the Type I NKT TCR test case 3HUJ (Figure 1), we initialized separate docking simulations, in addition to the original “start1” docking start site, from two alternative sites closer to the bound orientation to determine whether they would lead to TCRFlexDock hits. We employed a “start2” site that was approximately equidistant from the 3HUJ and 4MNG bound orientations, with a 20 Å translational shift along the CD1d helices from “start1” (see Methods), as well as a “fit” site that entailed root-mean-square fitting the unbound TCR to the bound TCR position (Figure 4). Despite substantial distance from the bound orientation (13.65 Å ligand RMSD), docking from the start2 site led to highly ranked hits using both ZR2 and ZRT scoring functions for 3HUJ (top hits ranked 10 and 8, respectively; Additional file 3: Table S1), though some non-hits from start1 docking ranked higher than these hits (Figure 4). Hits were not improved for test case 4MNG, indicating as noted above that the interface characteristics rather than the docking orientation alone likely caused the docking search to yield few favorable hits. As expected from their relatively large distances to the bound conformation (>18 Å ligand RMSD), for the remaining three cases the start2 docking start site led to no hits (Additional file 3: Table S1). When pooling results from start1 and start2 sites however, the near-native predictions were predominantly scored better by ZRT for all these cases (Additional file 4: Figure S3). For one test case (4LHU) we also tested pooling results from two additional start sites (one intermediate site between start1 and start2, and an extreme start site past start2), and ZRT continued to rank the near-native models best (Additional file 5: Figure S4).

### Prediction of TCR complexes with CD1b-GMM and CD1b-MA

Recently the unbound crystal structures of two clones of germline mycolyl-lipid reactive (GEM) αβ TCRs were reported [24]: clone 18 (PDB code 4G8E), which binds CD1b-MA, and clone 42 (PDB code 4G8F) which binds CD1b-GMM. As the complex structures have not been reported, and the structure of CD1b-GMM was reported in a previous study [25], we used TCRFlexDock to predict these two structures representing this class of interactions. As the α chains are nearly identical between the two GEM TCR clones and likely dominate the interactions with CD1b-Ag [24], we computed the distances between the models ranked in the top 20 by ZRT for the two TCRs based on the RMSDs between their germline CDRα loop positions (with CD1b superposed; Additional file 6: Figure S5). We found that the 4G8E and 4G8F models (for brevity, we refer to unbound TCR PDB codes to represent these two complexes) were highly similar to each other, with many of the top 20 models less than 4 Å apart. 4G8E model 3 and 4G8F model 12 exhibited the highest similarity in germ line CDRα positioning (1.37 Å RMSD) over CD1b-Ag, and fell into the largest cluster of models in both sets of top 20 predictions (the bottom left cluster in Additional file 7: Figure S6). On this basis, as well as notable contacts with antigen as described below, we selected these models for further analysis.

**Figure 5:**
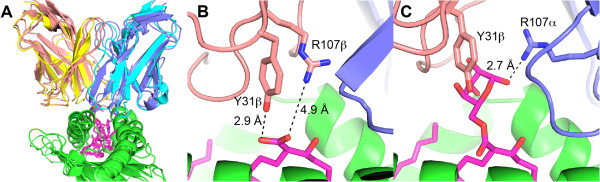
**Predictions of GEM TCR complexes with CD1b-Antigen. (A)** GEM TCR models bound to CD1b-Ag and Yae62 TCR bound to H-2 K^b^-peptide (PDB code 3RGV). Colors are: GEM α chains, blue; GEM β chains, salmon; Yae62 α chain, cyan; Yae62 β chain, yellow; CD1b/MHC, green; antigens MA/GMM/peptide, magenta. **(B)** Clone 18 GEM TCR modeled interface with CD1b-MA. **(C)** Clone 42 GEM TCR modeled interface with CD1b-GMM. For **(B)** and **(C)**, key TCR side chains contacting antigen head group and inter-atomic distances are indicated.

The structures of these models (Figure 5) provide a view of their overall recognition mode as well as several differences in TCR contacts mediating differential antigen recognition. GEM TCR engagement of CD1b is similar to characterized structures of αβ TCR recognition of pMHCs, including the murine Yae62 TCR bound to H-2 K^b^ and peptide (Additional file 7: Figure S6 and Figure 5A). Closer examination of the modeled interfaces shows key roles for certain TCR residues in mediating antigen recognition, for instance Tyr31β is positioned close to the antigen in both complexes. Arg107β of the 4G8E TCR is of particular interest, due to its potential electrostatic interaction with the negatively charged head group of the MA antigen (Figure 5B); this residue was also highlighted based on the unbound clone 18 structure as it represented a notable difference between the two clones’ CDR3 architectures [24]. While these models provide a likely mechanism of CD1b-Ag engagement, other sets of 4G8E and 4G8F models were identified that shared docking orientations with TCR-pMHC and MAIT-MR1-Ag interfaces (Additional file 7: Figure S6 and Additional file 8: Figures S7).

## Discussion

We have demonstrated that our TCR docking algorithm, TCRFlexDock, is capable of producing accurate models of TCRs recognizing MHC-like molecules presenting a variety of non-peptide ligands. By retraining our scoring function, we were able to improve the re-ranking performance for test cases representing this class of complexes. Further improvements, for instance to improve the ranking of hits for the 4MNG or 3HUJ cases and performance using pooled results from alternate docking start sites, could potentially be achieved through inclusion of other terms (e.g. long-range partial charge rather than full charge electrostatics), and further optimization of the pair potential parameters and antigen atom typing. Additionally, though antigen bond torsion angles were minimized during Rosetta docking simulations, explicit inclusion of lipid head group rotamers (based on pre-generated conformers) could represent another route for improving docking performance.

We report models of CD1b-bound GEM TCRs from TCRFlexDock that account for a likely shared docking orientation as well fine differences in antigen recognition. The docking searches for these cases were focused on the CD1b site analogous to the pMHC interface engaged by most TCRs; while outlier TCR binding modes have been observed for some non-MHC ligands [26] and would reduce change of docking success (as seen for test case 3HUJ), mutagenesis evidence strongly suggests that TCRs engage CD1b-Ag with a footprint similar to TCR-pMHC interactions [27]. Two previously reported TCR-CD1b-Ag modeling attempts (using other TCR clones) were based on homology with TCR-pMHC complex structures [25, 28], rather than a flexible docking search; notably, in one of these studies the TCR loops exhibited considerable clash with antigen in the modeled complexes [25], a challenge that is explicitly addressed during the conformational search of TCRFlexDock. Within our set of TCR-CD1b-Ag models, the dominant cluster was similar to the Class I MHC-bound Yae62 TCR (Figure 5 and Additional file 7: Figure S6). However, we did identify alternative models, including several that were similar to the TRBV6 MAIT TCR-MR1-Ag and ELS4 TCR-HLA-peptide complexes. As noted by others [24], the MAIT, ELS4 and GEM TCRs share TRAV1-2 genes and the former two exhibit similar docking orientations over their respective ligands [8], yet given the structural and residue-level differences between CD1b, MR1 and MHC, there is a significant possibility that TCR-CD1b-Ag interactions exhibit a distinct binding conformation, as we identified using TCRFlexDock. Experimental structural characterization of GEM TCR-CD1b-Ag complexes will allow determination of which models are most accurate.

While this manuscript was under review, the x-ray structure of a TRBV20-containing MAIT TCR (corresponding to the unbound TCR we used for test case 4L4T) in complex with MR1 and antigen was reported [29]. Though the antigens of our docking simulation and the solved structure are not identical (thus preventing an analysis of native contacts and hits), we evaluated interface backbone RMSD between our models and the solved structure (PDB id 4PJ8), and the top-ranked model by ZRT score indeed had a low interface RMSD with respect to the experimentally determined complex structure (1.68 Å; Additional file 9: Figure S8).

## Conclusions

These updates to the TCRFlexDock algorithm, as well as the docking test cases we assembled, represent an advance in modeling TCR-ligand recognition, extending capabilities beyond TCR-pMHC modeling methods reported by ourselves [11] and others [30, 31] to modeling complexes with lipid and metabolite antigens presented by MHC-like molecules. Our models of MAIT TCR-MR1-Ag and GEM TCR-CD1b-Ag complexes, which have not been described crystallographically, provide likely mechanisms of ligand engagement by these TCRs, and highlight the ability of advanced protein docking algorithms to complement experimental techniques in probing the structural basis of molecular recognition. These methods can be applied to model other TCRs bound to MHC-like ligands, as more of these interactions and TCRs become characterized, such as a γδ TCR clone that was recently found to engage the endothelial protein C receptor [32].

## Methods

### Input structures and TCR docking

TCR docking test cases were identified based on identical sequences between TCRs and ligands from unbound and bound PDB entries. Lipid and metabolite antigens were also evaluated for unbound and bound structural matches. For test case 4LHU, the lipid molecules were not identical (JLS for the bound CD1d-α-GalCer, AGH for the unbound CD1d-α-GalCer) but this was due to differences in buried hydrocarbon chains, while the exposed head groups were the same. Prior to docking, missing unbound TCR CDR loop residues were added to PDB files 4LFH (test case 4LHU), 4MNH (test case 4MNG), 4G8E, and 4G8F using Modeller [33], keeping the remainder of the TCR fixed. The unbound CD1b-MA structure was generated by truncation of the GMM antigen in the unbound CD1b-GMM structure (PDB code 1UQS).

Flexible TCR docking was performed in the same manner as presented previously [11], using a modified version of RosettaDock to generate 1000 docking models starting with unbound structures in a predetermined starting orientation (described in more detail below), followed by re-scoring docking models using ZRANK. RosettaDock simulations were performed using an iterative procedure (written with RosettaScripts) where the CDR3 loops undergo a perturbation using the kinetic closure (KIC) loop modeling protocol in Rosetta, followed by Monte Carlo-based sampling of local rigid-body docking orientation, side chain rotamer optimization, and two rounds of KIC refinement of all CDR loops. Parameters for lipid and antigens were generated using Open Babel [34] to convert from PDB to Mol2 format, followed by conversion to Rosetta parameter files using the molfile_to_params.py script [35]. This process was also used to determine antigen torsion angles that were minimized by Rosetta during the docking process. Docking was performed on a Linux cluster with 2.8 GHz AMD Opteron cores; producing 50 docking models on a single core (20 such jobs were run in parallel for each test case) took approximately 10 hours on average (12 minutes per model). For ZRANK, antigen partial charge parameters were obtained from polar hydrogen Mol2 files from Open Babel, while IFACE and ACE atom types were assigned based on congruence with amino acid atoms.

### Initial and alternate docking input positioning

As with our previous TCR docking study, for initial docking start position (corresponding to the “start1” site), TCRs were aligned with pseudo-symmetric axis perpendicular to the plane of the MHC-like helices, positioned over the MHC-like helix centroid at a distance of 25 Å, with a 45° crossing angle. We also tested an alternate “start2” docking start site; for this site, with respect to “start1”, the TCR was shifted by 20 Å along the MHC-like helix axis toward the α1 helix C-term, rotated to a 0° crossing angle, and tilted by 25° toward the MHC-like helix axis C-term. Additional “start3” and “start4” sites included 10 Å and 25 Å TCR shifts, crossing angles of 22.5° and -15°, and tilts of 12.5° and 40°, respectively.

### Evaluation of docking models

Docking models were evaluated using CAPRI criteria to assess predictions as “incorrect”, “acceptable”, “medium accuracy” and “high accuracy” [21]. As with our previous TCR docking study, models assessed as medium or high accuracy are referred to as hits. Lipid and metabolite antigen atoms were included in calculation of bound contacts with TCR residues, but were omitted from RMSD calculations (only protein backbone atoms were used).

### Retraining ZRANK scoring function

As for the original ZRANK implementation [36], we employed a downhill simplex to select weights for the terms of the ZRANK scoring function, in this case maximizing the average area under the receiver operating characteristic curve (AUC) of 20 TCR-pMHC docking test cases [11], distinguishing hit from non-hit predictions among the top 30 ranked models for each TCR-pMHC case. To avoid local minima, the downhill simplex was run from 12,000 randomly generated starting points. For three-fold cross-validation within the TCR-pMHC set, training and testing sets were selected such that cases with the same TCR were not used simultaneously in both sets. The retrained scoring function weights (and ZR2 weight values [22] in parentheses, for comparison) used to select TCR docking models are:

- van der Waals attractive: 0.027 (1.0)
- van der Waals repulsive: 1.33 (0.23)
- Electrostatics short-range attractive: 0.35 (0.57)
- Electrostatics short-range repulsive: 0.29 (0.56)
- Electrostatics long-range attractive: 0.30 (1.09)
- Electrostatics long-range repulsive: 0.21 (0.29)
- ACE: 0.64 (0.7)
- IFACE: 0.27 (0.38)

### Figures

Plots were generated using gnuplot (http://www.gnuplot.info), and molecular visualizations were produced using PyMOL (http://www.pymol.org). Clustering and generation of the unrooted tree were performed using the APE package [37] in R (http://www.r-project.org).

## Electronic supplementary material

Additional file 1: Figure S1: Receptor interface RMSD versus ligand RMSD for test cases 4EI5 and 4LHU. (PDF 150 KB)

Additional file 2: Figure S2: Top-ranked ZRT model for test case 4EI5, showing (A) complex and (B) α chain interface with CD1d-Ag. CD1d is green, crystal structure TCR α and β chains are slate and tan, predicted TCR α and β chains are orange and blue, unbound Ag is magenta, bound Ag is cyan, and predicted Ag is yellow. (PDF 1014 KB)

Additional file 3: Table S1: Docking performance when initiated from the “start2” site. (DOCX 14 KB)

Additional file 4: Figure S3: ZRT score versus interface RMSD for docking test cases. Red circles represent models from the “start1” docking start site, while blue triangles represent models from the “start2” docking start site. (PDF 418 KB)

Additional file 5: Figure S4: ZRT score versus interface RMSD for docking test case 4LHU, using four docking start positions. Docking start sites shown are “start1” (red circles), “start2” (blue triangles), “start3” (magenta triangles), and “start4” (cyan circles). (PDF 251 KB)

Additional file 6: Figure S5: Distances between top 20 models (ranked by ZRT score) of Clone 18 GEM TCR (4G8E) bound to CD1b-MA and models of Clone 42 GEM TCR (4G8F) bound to CD1b-GMM, calculated using shared (identical in sequence) CDR1α and CDR2α loops. The lowest RMSD among all pairs of models (1.37 Å) is boxed, and corresponds to the predictions selected for further analysis (4G8E model 3 and 4G8F model 12). (PDF 55 KB)

Additional file 7: Figure S6: RMSD-based clustering of the top 20 4G8E and 4G8F models bound to CD1b-Ag. Selected sets of models are circled to indicate similarity with existing TCR complex crystal structures based on comparison of variable domain orientations after superposition of MHC or MHC-like structures. (PDF 37 KB)

Additional file 8: Figure S7: Additional GEM TCR-CD1b-Ag models compared with TCR-pMHC structures. Shown are (A) 4G8E-CD1b-MA model 18 and 1G4 TCR-HLA-A2-peptide (PDB code 2BNR), and (B) 4G8F-CD1b-GMM model 5 and TRBV6 MAIT TCR-MR1-6FP CD1b-Ag (PDB code 4L4V). Colors are: GEM TCR α chains, blue; GEM TCR β chains, salmon; crystallographic α chain, cyan; crystallographic β chain, yellow; CD1b/MR1/MHC, green; antigens, magenta. (PNG 635 KB)

Additional file 9: Figure S8: ZRT score versus interface RMSD for the 4L4T test case models (from the original “start1” docking position) evaluated against the recently released crystal structure of the same MAIT TCR in complex with a distinct Ag and MR1 (PDB code 4PJ8). (PDF 113 KB)
