# Supplementary figures and images for: Modeling T cell receptor recognition of CD1-lipid and MR1-metabolite complexes
Source: BMC Bioinformatics. 2014 Sep 26;15(1):319. doi: 10.1186/1471-2105-15-319 (PMC4261541; doi:10.1186/1471-2105-15-319)

4EI5

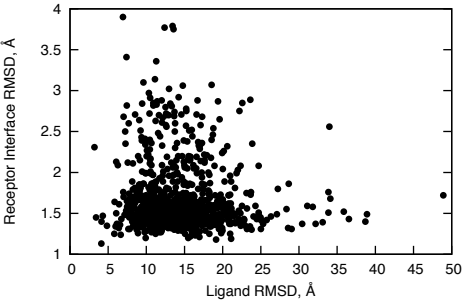

4LHU

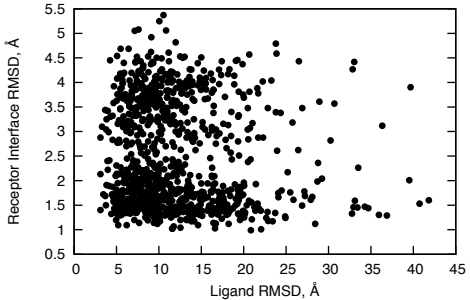

Supplement: Supplementary file 1 — Additional file 1: Figure S1: Receptor interface RMSD versus ligand RMSD for test cases 4EI5 and 4LHU. (PDF 150 KB) [file 12859_2014_6632_MOESM1_ESM.pdf]

A

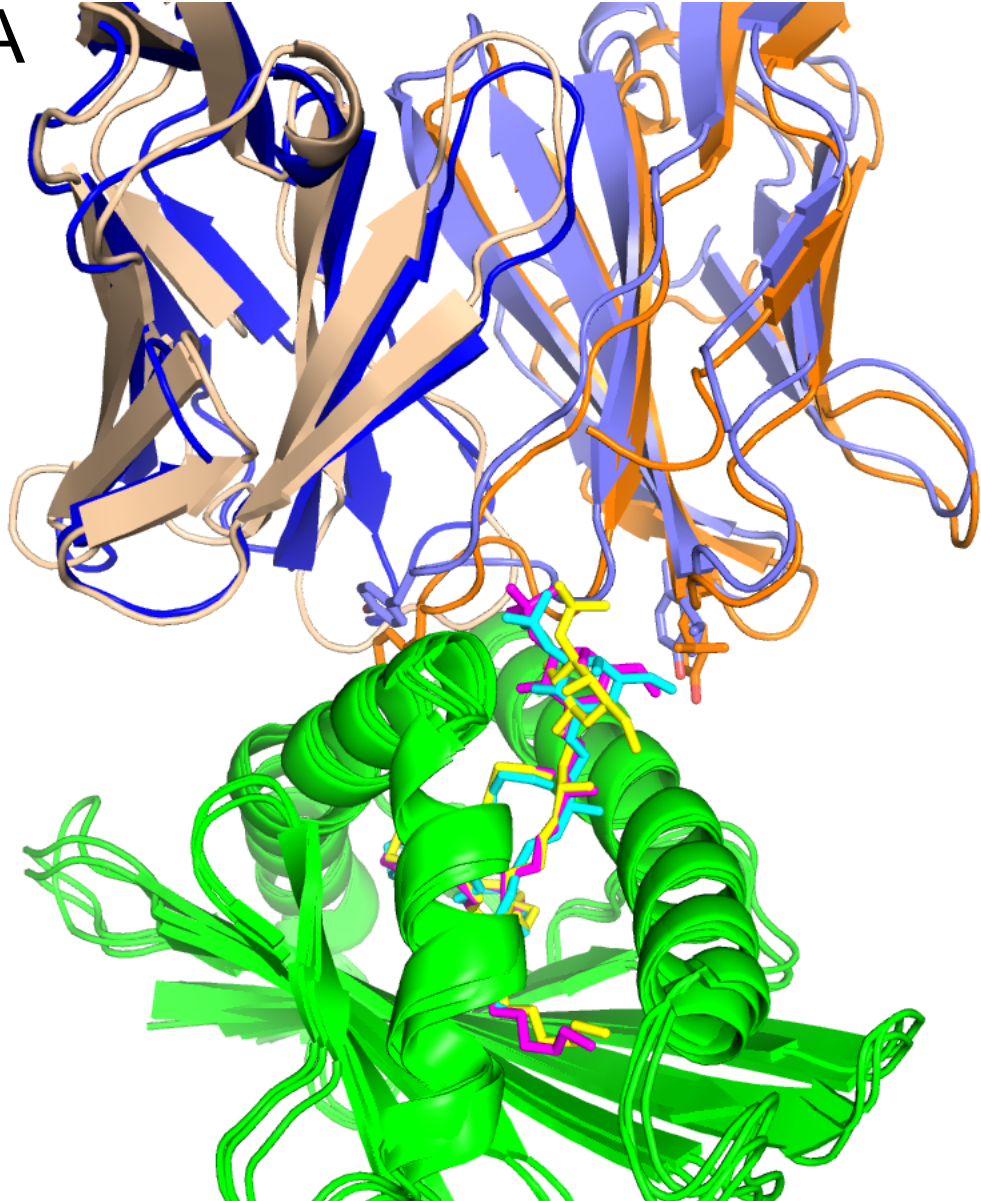

B

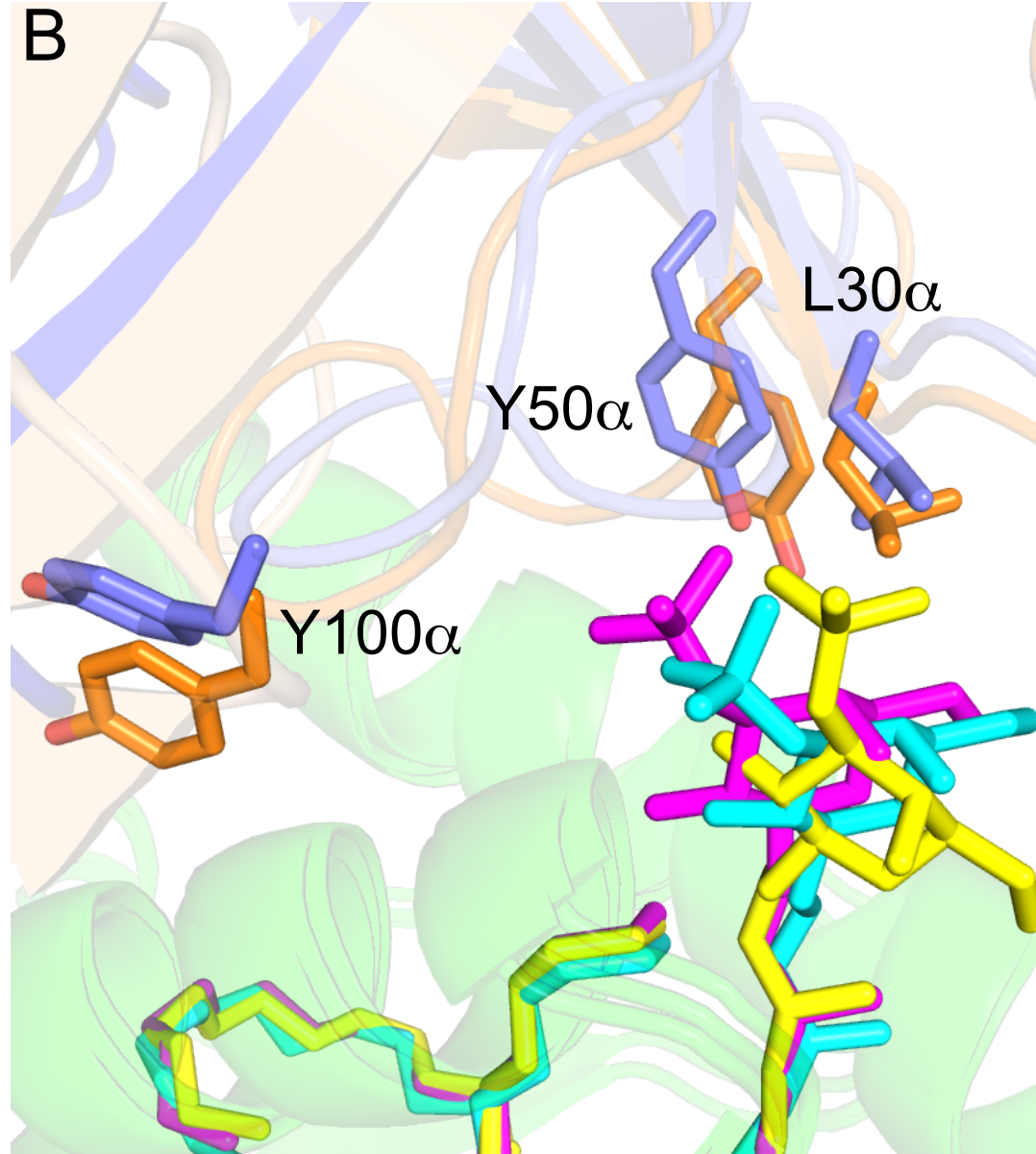

Supplement: Supplementary file 2 — Additional file 2: Figure S2: Top-ranked ZRT model for test case 4EI5, showing (A) complex and (B) α chain interface with CD1d-Ag. CD1d is green, crystal structure TCR α and β chains are slate and tan, predicted TCR α and β chains are orange and blue, unbound Ag is magenta, bound Ag is cyan, and predicted Ag is yellow. (PDF 1014 KB) [file 12859_2014_6632_MOESM2_ESM.pdf]

4EI5

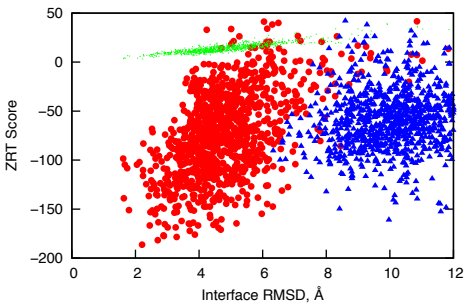

4L4T

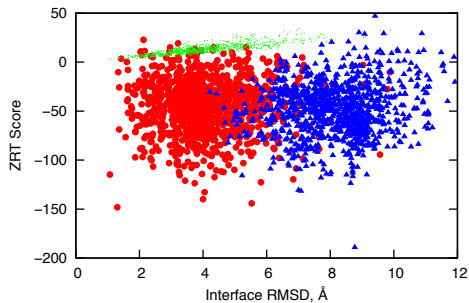

4LHU

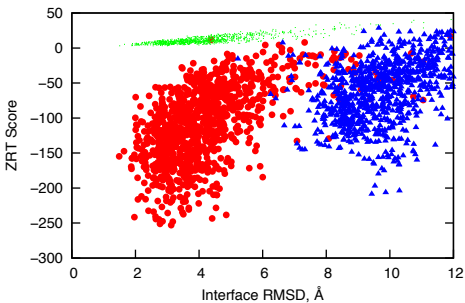

4MNG

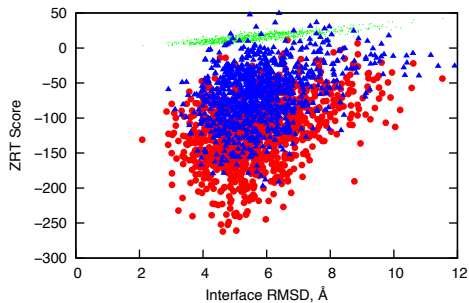

Supplement: Supplementary file 4 — Additional file 4: Figure S3: ZRT score versus interface RMSD for docking test cases. Red circles represent models from the “start1” docking start site, while blue triangles represent models from the “start2” docking start site. (PDF 418 KB) [file 12859_2014_6632_MOESM4_ESM.pdf]

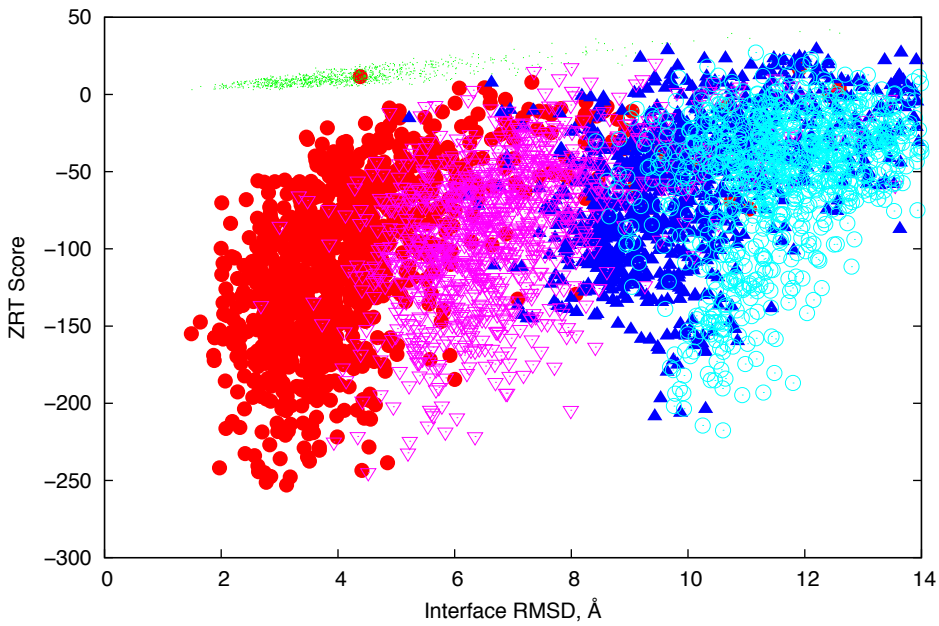

Supplement: Supplementary file 5 — Additional file 5: Figure S4: ZRT score versus interface RMSD for docking test case 4LHU, using four docking start positions. Docking start sites shown are “start1” (red circles), “start2” (blue triangles), “start3” (magenta triangles), and “start4” (cyan circles). (PDF 251 KB) [file 12859_2014_6632_MOESM5_ESM.pdf]

GEM TCR Complex CDR $\alpha$  RMSDs

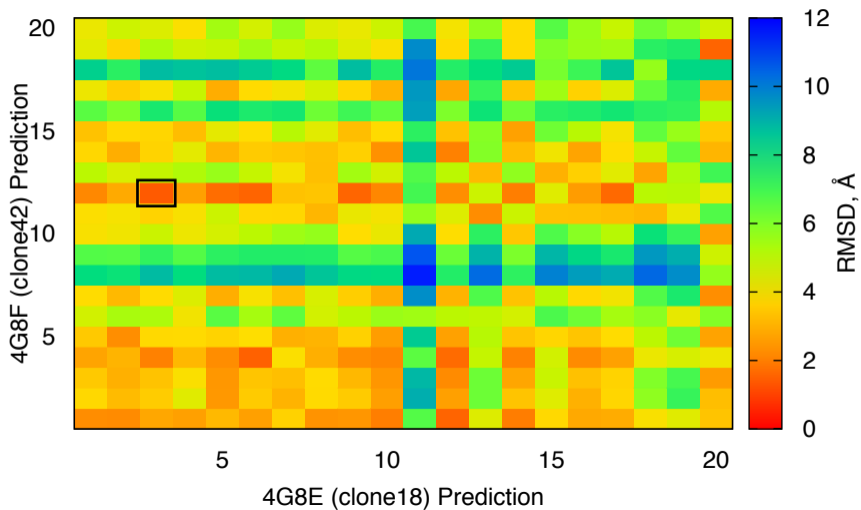

Supplement: Supplementary file 6 — Additional file 6: Figure S5: Distances between top 20 models (ranked by ZRT score) of Clone 18 GEM TCR (4G8E) bound to CD1b-MA and models of Clone 42 GEM TCR (4G8F) bound to CD1b-GMM, calculated using shared (identical in sequence) CDR1α and CDR2α loops. The lowest RMSD among all pairs of models (1.37 Å) is boxed, and corresponds to the predictions selected for further analysis (4G8E model 3 and 4G8F model 12). (PDF 55 KB) [file 12859_2014_6632_MOESM6_ESM.pdf]

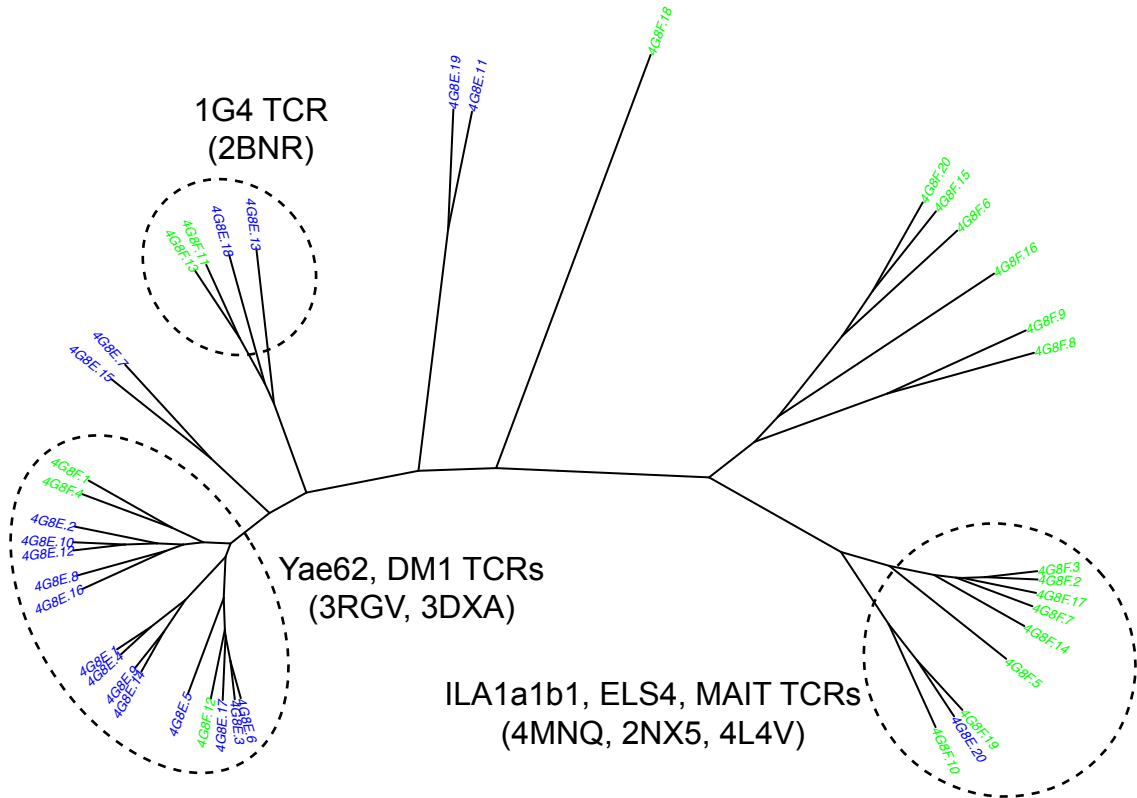

Supplement: Supplementary file 7 — Additional file 7: Figure S6: RMSD-based clustering of the top 20 4G8E and 4G8F models bound to CD1b-Ag. Selected sets of models are circled to indicate similarity with existing TCR complex crystal structures based on comparison of variable domain orientations after superposition of MHC or MHC-like structures. (PDF 37 KB) [file 12859_2014_6632_MOESM7_ESM.pdf]

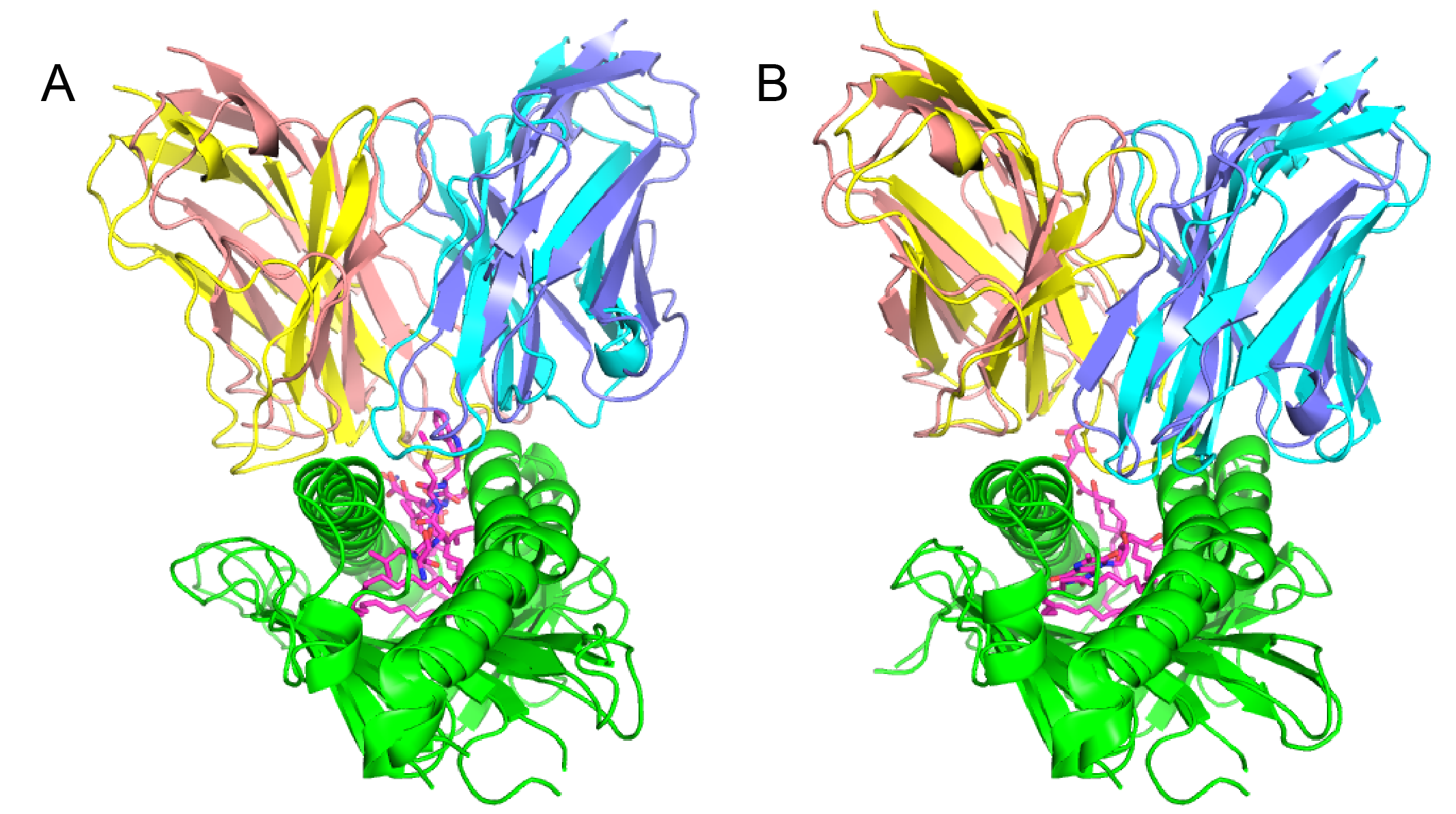

Supplement: Supplementary file 8 — Additional file 8: Figure S7: Additional GEM TCR-CD1b-Ag models compared with TCR-pMHC structures. Shown are (A) 4G8E-CD1b-MA model 18 and 1G4 TCR-HLA-A2-peptide (PDB code 2BNR), and (B) 4G8F-CD1b-GMM model 5 and TRBV6 MAIT TCR-MR1-6FP CD1b-Ag (PDB code 4L4V). Colors are: GEM TCR α chains, blue; GEM TCR β chains, salmon; crystallographic α chain, cyan; crystallographic β chain, yellow; CD1b/MR1/MHC, green; antigens, magenta. (PNG 635 KB) [file 12859_2014_6632_MOESM8_ESM.png]

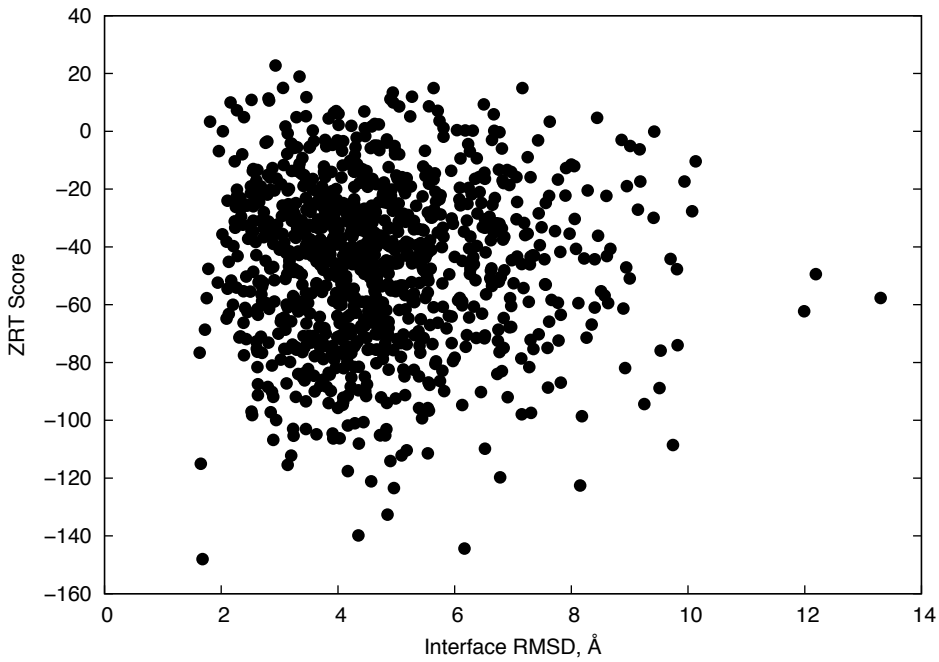

Supplement: Supplementary file 9 — Additional file 9: Figure S8: ZRT score versus interface RMSD for the 4L4T test case models (from the original “start1” docking position) evaluated against the recently released crystal structure of the same MAIT TCR in complex with a distinct Ag and MR1 (PDB code 4PJ8). (PDF 113 KB) [file 12859_2014_6632_MOESM9_ESM.pdf]
