# Supplementary material for: Modeling T cell receptor recognition of CD1-lipid and MR1-metabolite complexes
Source: BMC Bioinformatics. 2014 Sep 26;15(1):319. doi: 10.1186/1471-2105-15-319 (PMC4261541; doi:10.1186/1471-2105-15-319)
Supplement: Supplementary file 3 — Additional file 3: Table S1: Docking performance when initiated from the “start2” site. (DOCX 14 KB) [file 12859_2014_6632_MOESM3_ESM.docx]

Additional file Table S1. Docking performance when initiated from the “start2” site.

|  | **Start RMSD, Å** | |  |  |  |  |
| --- | --- | --- | --- | --- | --- | --- |
| **Test Case** | **Ligand** | **Interface** | **ZR2 Rank^1^** | **ZRT Rank^1^** | |  |
| 3HUJ | 13.65 | 4.55 | 11 | 8 | |  |
| 4EI5 | 21.01 | 10.11 | - | - | |  |
| 4LHU | 18.72 | 9.71 | - | - | |  |
| 4MNG | 14.84 | 4.64 | 763 (10) | 554 (8) | |  |
| 4L4T^2^ | 20.2 | 8.11 | - | - | |  |

^1^Rank of the first hit; for 4MNG, values in parentheses denote the ranks of the first “acceptable” prediction.

^2^Only TCR α chain and MR1 were used to evaluate these predictions, as unbound and bound TCR β chains differ in sequence.
